# Forest dynamics in the U.S. indicate disproportionate attrition in western forests, rural areas and public lands

**DOI:** 10.1371/journal.pone.0171383

**Published:** 2017-02-22

**Authors:** Sheng Yang, Giorgos Mountrakis

**Affiliations:** Department of Environmental Resources Engineering, State University of New York, College of Environmental Science and Forestry, Syracuse, New York, United States of America; Technion Israel Institute of Technology, ISRAEL

## Abstract

Forests are experiencing significant changes; studying geographic patterns in forests is critical in understanding the impact of forest dynamics to biodiversity, soil erosion, water chemistry and climate. Few studies have examined forest geographic pattern changes other than fragmentation; however, other spatial processes of forest dynamics are of equal importance. Here, we study forest attrition, the complete removal of forest patches, that can result in complete habitat loss, severe decline of population sizes and species richness, and shifts of local and regional environmental conditions. We aim to develop a simple yet insightful proximity-based spatial indicator capturing forest attrition that is independent of spatial scale and boundaries with worldwide application potential. Using this proximity indicator, we evaluate forest attrition across ecoregions, land ownership and urbanization stratifications across continental United States of America. Nationally, the total forest cover loss was approximately 90,400 km^2^, roughly the size of the state of Maine, constituting a decline of 2.96%. Examining the spatial arrangement of this change the average FAD was 3674m in 1992 and increased by 514m or 14.0% in 2001. Simulations of forest cover loss indicate only a 10m FAD increase suggesting that the observed FAD increase was more than an order of magnitude higher than expected. Furthermore, forest attrition is considerably higher in the western United States, in rural areas and in public lands. Our mathematical model (R^2^ = 0.93) supports estimation of attrition for a given forest cover. The FAD metric quantifies forest attrition across spatial scales and geographic boundaries and assesses unambiguously changes over time. The metric is applicable to any landscape and offers a new complementary insight on forest landscape patterns from local to global scales, improving future exploration of drivers and repercussions of forest cover changes and supporting more informative management of forest carbon, changing climate and species biodiversity.

## Introduction

Forests are a critical natural resource serving multiple purposes and a wide range of stakeholders. The act of forest cover removal, known as deforestation, for example for agricultural, forest harvesting or oil extraction purposes [1], is extensively studied from numerous perspectives including biodiversity [2–4], carbon sequestration [5], water quality [6], biogeochemical cycles [7–9], and forest resource sustainability [10]. Over 30% of the conterminous U.S. is covered by forestlands [11], and U.S. forests have been affected by dramatic land cover change over the past few centuries [12]. With the proliferation of satellite-derived observations, large-scale deforestation studies have been feasible since 1972 [13] leading to a significant quantity of deforestation research [14–22]. Forest disturbances can result in a variety of forest spatial patterns with substantially different impacts on ecological functions and processes [23–26], but methods of quantifying these differences over space and time have been lacking.

Numerous geographic indicators have been proposed but none supports multi-temporal comparisons focusing on the processes of forest dynamics, mostly forest attrition, at various spatial scales independent of study boundaries while allowing intuitive understanding by non-experts [27–29]. When using existing indicators, mostly landscape indices that evaluate forest landscape patterns at one snap shot of time, the multi-temporal comparisons of their values can often be problematic because of their low sensitivity and non-uniqueness in differentiating types and magnitudes of changes in spatial patterns between two or more time points [30–33]. Most existing studies utilizing fragmentation metrics measure only one-time snapshot of forest landscape pattern without comparison among multiple times [34–36]. A few studies which included multiple time points concentrated on the comparisons between two or more static conditions but lacked indicators focusing on the process of fragmentation [37,38]. In addition, few studies have examined forest geographic patterns other than fragmentation; however, other spatial processes of forest dynamics are of equal importance [39,40]. Here, we concentrate on forest landscape spatial transformation processes over time and specifically on forest attrition, the removal of clustered and isolated forest patches. The disappearance of entire forest patches can cause significant changes in biodiversity [41,42]. Large patches in a more pristine condition are irreplaceable for conserving biodiversity and the attrition of these patches is especially problematic [43]. Stand-removing disturbances of forest patches account for a major part of decreasing forest carbon sequestration, changing climate, and species extinction [44–46]. The relative importance of multi-scale drivers affecting species diversity in small forest patches was investigated, with highest variation explained by patch-size indicators [47]. The non-random pattern of forest attrition, i.e., complete losses of larger or interior forest patches, can greatly impact seasonal annual precipitation and diurnal range of temperatures in nearby forests [41]along with intra- and inter-continental climate [48].

Due to their spatial arrangement isolated forest patches are disproportionally exposed to edge effects. The forest edges interact with adjacent land covers/uses which could result in higher invasive species exposure [49,50]. In addition, anthropogenic pressures are more pronounced on edges via road establishment [51,52], agricultural intentional or natural expansion [53,54], and soil compaction via recreational activities [55]. For example, it has been shown that understory plant species composition is limited in forest edges [56]. Despite the increased pressures imposed on smaller forest patches it has been argued that their conservation is worthy as when these small forest patches are uniformly distributed they could enhance inter-patch habitat diversity as opposed to large forest patches [56–58].

In light of the importance of forest attrition and the lack of research on this topic, we propose an intuitive yet expressive metric, the forest attrition distance (FAD). The FAD metric is primarily based on the average of distances from non-forest and forest areas to nearest forests in a landscape. Higher FAD values at one snapshot of time are indicative of larger patches of non-forested areas. The metric is consistent with the ‘habitat amount hypothesis’ [59]by examining the patch isolation effect and its surrounding local landscape. From the biodiversity perspective patch isolation has been found to be an important indicator for species richness [60–62]. Although there are two studies which mentioned metrics, i.e., GISfrag and Mean distance to Forest Edge, with similar distance calculation [38,63], FAD offers significant differences. First, the application of FAD is different from the other two. FAD was applied on continental scales in the U.S. with a large variation in size and boundaries of study areas compared with GISfrag which was applied on local scales. FAD calculation includes distances of both forest and non-forest pixels to forests, not just forest pixels within forests in [38], therefore it examines the evolution of the entire landscape focusing on forest attrition. The application of FAD includes normalization to account for the nonlinear relation between forest cover and FAD, which is critical in interpreting results unambiguously. The application also includes statistical simulations under different forest cover as benchmarks to support statistically rigorous interpretation. These are not included in the other two metrics. FAD doesn’t require delineation of managed forest patch [63] or fixed 1km cells as aggregation unit [38]. Instead, FAD is based on forest definition consistent with the one in United Nationals Framework Convention on Climate Change (UNFCCC) and ecological regions are used as aggregation units, which is more meaningful ecologically. The FAD metric offers several conceptual and practical advantages thus closing an important knowledge gap: i) it is independent of artificial geographic boundaries as the nearest forest could be found in an adjacent area, ii) it is conceptually independent of spatial scale and extent, as it can be applied from meters to kilometers and from local to global studies, iii) it unambiguously assesses changes over time, and iv) it is simple to communicate to non-experts.

In the forthcoming sections we first show the ability of the FAD indicator to capture forest attrition, then we proceed with a case study in the conterminous U.S. More specifically, we investigate forest attrition during the 1990s and investigate statistical discrepancies across urbanization levels and ownership types. Looking beyond the U.S., global tree cover maps have been produced recently expressing forest extent, loss, and gain from 2000 to 2012 [64]. The 30m spatial resolution along with the global coverage provide a unique opportunity to study forest attrition at unprecedented spatial extents. Our method is directly applicable to this global dataset, where important worldwide spatial patterns could be revealed benefiting future research of drivers and consequences of forest spatial pattern changes.

## Materials and methods

### 2.1 Attrition metric calculation and applicability illustration

The FAD metric is computed as the average of distances from non-forest and forest areas to nearest forests in a landscape. Higher FAD values are indicative of larger patches of non-forested areas. Similar proximity metrics have been used in road ecology to assess landscape changes by road encroachments in the U.S. [65]. FAD encapsulates the levels of forest landscape transformation consistent with the classic model [39] and quantifies the magnitude of forest attrition over time.

Forest attrition distance requires a binary forest image to operate. When a pixel is forested the FAD value is zero. The FAD value of every pixel is then aggregated over an arbitrary spatial polygon (e.g. ecoregion, state, county) to assign an average FAD value. A higher average FAD value indicates larger gaps of non-forest areas suggesting higher level of forest fragmentation and loss corresponding to later stages of forest landscape transformation [39].

**Fig 1:**
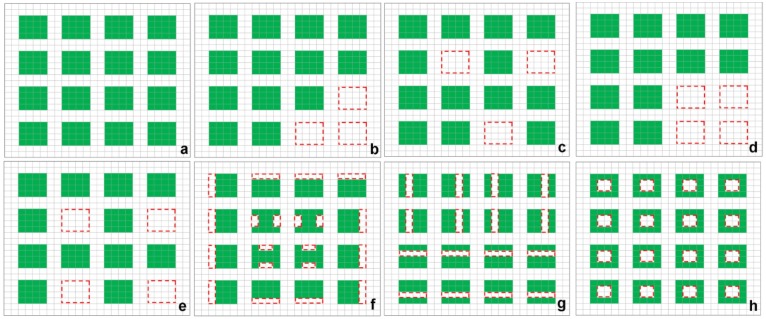
Illustration of applicability of the forest attrition distance metrics used as an attrition indicator. Case a shows the initial forest state followed by seven forest change examples (b-h). Forested area is depicted in green pixels. Red squares with dotted lines indicate forest loss. Values of our forest attrition distance along with four other commonly used metrics for these seven cases are provided in Table 1.

The advantage of FAD over other commonly used habitat fragmentation/isolation metrics is that FAD can consistently relate to spatial patterns of forest landscape changes, namely perforation, subdivision, fragmentation, shrinkage, and attrition [39] (Fig 1 and Table 1). We combined fragmentation and subdivision as one category named subdivision due to the similarity between the two. The starting forest state is presented in Fig 1a followed by seven examples of forest change (cases b through h). Cases b and Case c show the same amount of forest attrition loss (-18.75%). Case b has considerably higher FAD than Case c due to more clustered clear-cutting forest losses or attrition (see Table 1 for values). Similarly, Case d has higher FAD than Case e despite the same amount of patch attrition (-25%), because Case d experienced more clustered forest losses. Cases f has the same amount of forest loss as Case d and e (-25%), but the FAD increase in this case is much smaller due to more scattered forest loss of early stages of landscape transformation which is characterized by shrinkage of forest patches comparing to complete external losses from attrition in Case d and e. Finally, cases g and h have smallest FAD increase (52.2% and 48.6%) given the same forest loss (-25%) as case d, e, and f, and these two cases represent the earliest phases of landscape transformation, namely subdivision and perforation with scattered forest losses occurring internally in forest patches. For comparison purposes four commonly used metrics are also calculated for the seven scenarios and presented in Table 1. Results indicate that only our metric can differentiate between all seven scenarios.

**Table 1:** FAD and commonly used fragmentation metrics in seven cases of forest landscape transformation.

Metrics	a. Original forest	b. Aggregated attrition	c. Dispersed attrition	d. Aggregated attrition	e. Dispersed attrition	f. Shrinkage	g. Subdivision	h. Perforation
FC (m^2^)	230400	187200(-18.75%)	187200(-18.75%)	172800(-25%)	172800(-25%)	172800(-25%)	172800(-25%)	172800(-25%)
FAD (m)	24.7	49.6(100.8%)	35.8 (44.9%)	63(155.1%)	42.4(71.7%)	34.7(40.5%)	28.2(14.2%)	27.6(11.7%)
FAD/FC (km/km^2^)	72.6	179(146.6%)	129.3(78.1%)	246.5(239.5%)	165.7(128.2%)	135.6(86.8%)	110.5(52.2%)	107.9(48.6%)
MNND (m)	60	60	60	60	60	60	30 (-50%)	60
PI	18	15.4(-14.4%)	9.6(-46.7%)	14.3(-20.6%)	11(-38.9%)	12.4(-31.1%)	12.5(-30.6%)	12.4(-31.1%)
AWMP (m^2^)	14400	14400	14400	14400	14400	10800(-25%)	6000(-58.3%)	10800(-25%)
ED (km/ha)	0.33	0.33	0.33	0.33	0.33	0.43 (30.3%)	0.35 (6.1%)	0.67 (103%)

FC, Forest Cover; MNND, Mean Nearest Neighbor Distance; PI, Proximity index; AWMP, Area-weighted mean patch size; ED, Edge Density.

Values in parentheses are percentage changes compared with original forest in case a.

### 2.2 Creation of multi-temporal forest binary maps

Forest change binary maps were based on the National Land Cover Database (NLCD) 1992/2001 Retrofit Land Cover Change Product. The change product was developed by the U.S. Geological Survey to support better comparisons between NLCD 1992 [66] and NLCD 2001 [67]. The product contains land cover classes in Anderson Level I, including open water, urban, barren, grassland/shrub, agriculture, wetlands, and ice/snow, and all observed “from-to” classes, such as wetland-to-forest and forest-to-agriculture [68]. The retrofitted change product provided an appropriate direct comparison between land cover in 1992 and in 2001 which would not have been accurate using original NLCD 1992 and NLCD 2001 data considering substantial differences between the two products [68]. NLCD 1992/2001 change product is the first data which support pixel-to-pixel comparison across U.S. over a decade and it for the first time unlocked valuable information in the period unavailable before the release of the data. In addition, the earlier period has large amount of data and documented research about forest ecology, biodiversity, biogeochemistry, carbon cycle, soil erosion, human health, etc., thus rich supporting information to help understand and interpret the patterns discovered in our study. Finally, most efforts of forest cover change have focused on recent change (2000-present) [64,69,70]. However, historical benchmarks are necessary to understand the causes and effects of these changes and to assess the effectiveness of land-use policies, e.g., Reducing Emissions from Deforestation and Degradation (REDD) [71,72]. The overall accuracy of forest cover changes for the retrofit change product is close to 80% [72]. To limit the impact of misclassifications of forest cover we upsampled the original product while we placed measures to remove isolated pixels. The first step towards the binary forest map creation involved the reclassification of the original datasets of the NLCD change product. Two binary forest maps including only forest and non-forest classes were generated for 1992 and 2001 at the 30m resolution. The forest class included deciduous forest, evergreen forest, and mixed forest, and the non-forest class contained the remaining classes. To address computational limitations of forest attrition distance calculations across the entire U.S. the 30m forest binary maps were resampled to 270m using the 1 ha as the minimum forest size which is suggested by UNFCCC in Kyoto Protocol as an international agreement to mitigate climate changes [73]. The values and interpretation of FAD are likely affected by the definition of forest and it’s the priority of this study to choose the most meaningful definition. In this definition the term “forest cover” represents a certain density of trees instead of land use definition in forestry [74]. The term indicates both binary (presence vs. absence) and continuous (e.g. percentage) scales of forest representation, thus relevant to ecosystem processes such as chemical (e.g. carbon) and hydrological cycling, biodiversity, energy budget [72]. The down-sampling was performed by overlaying a 9×9 mask at the 30m representation (9×30m = 270m) and if at least 11 pixels, (approximately1 ha), out of the possible 81 pixels underneath that mask belonged to the forest class, then that new 270m pixel was assigned as forest. This step also decreased the number of very small patches which may be caused by NLCD image classification errors. The 1 ha minimum forest definition is also tested in 450m pixel and it’s determined that 270m resolution has a more reasonable forest cover percentage threshold (11/(9*9) = 13.6%) for 1 ha forest compared with relatively small threshold in 450m resolution (11/(15*15) = 4.89%) considering the more reasonable forest density in the downscaled cells and thus more ecological meaningful interpretations of the results. The smaller resolution, e.g., 90m, was not tested, because the cell size is smaller or very close to the minimum forest size and not big enough to eliminate classification errors. The scale and location of forest cover changes does not bias the process because the resampling is conducted in both 1992 and 2001 with the same threshold.

A final filtering step took place at the 270m binary representation to eliminate isolated forest pixels that significantly affect forest attrition distance calculations, which serves to improve data/noise balance by decreasing the effects from noise pixels derived from classification error in 30m resolution and resampled to 270m resolution. An isolated forest pixel was defined as the only forest pixel in the square area of 9×9 270m pixels. A filtering mask with 9×9 pixels was applied to remove isolated forest pixels in an approximate 600 ha non-forest neighborhood area. If the center pixel of the mask was the only forest pixel in the 9×9 neighborhood, then it was converted to the non-forested class. The resulting datasets were two forest binary maps in 1992 and 2001 at 270m pixel size providing forest cover information across the continental U.S. These two maps were used exclusively in all experiments.

### 2.3 Urbanization and landscape ownership strata

The 2000 TIGER/Line files were used as data source for the masks of urban/urban cluster/rural based on population density [75]. The TIGER/Line files were extracts from the Census TIGER (Topologically Integrated Geographic Encoding and Referencing) database of selected geographic and cartographic information. In the 2000 Census, the Census Bureau classified as urban all territory located within urbanized areas (UAs) and urban clusters (UCs). The Census Bureau delineates UA and UC boundaries that represent densely developed territory, encompassing residential, commercial, and other non-residential urban land uses. UAs consist of areas of 50,000 or more people and UCs represents areas of at least 2,500 people but fewer than 50,000 people, resulting in a representation of the “urban footprint”. Rural consists of all territory, population, and housing units located outside UAs and UCs. These three categories of vector files were then converted to raster maps of 270m spatial resolution consistent with forest cover and forest attrition distance maps. The boundaries and classes of urbanization are shown in S1 Fig.

The dataset of land ownership information is derived from the Protected Areas Database (PAD) version 2 [76]. PAD provides detailed information of protected fee and easement lands, including the ownership categories of these lands. There are 6 classes of ownerships (with minor aggregation from original classes); (1) Federal Land; (2) State Land; (3) Local Land; (4) Private Land (5) Private—regulated (aggregated from Native American Land and Private-Protected Land) (6) Others (aggregated from Joint Ownership and Unknown land). The land ownership maps were generated by converting PAD data to 270m raster maps consisting of areas of public and private lands, and forest ownership was then determined by overlaying forest maps and the ownership raster maps (see S2 Fig). In other words, forest attrition is studied by stratifying forestlands based on Protected Areas Database (PAD) and comparing forest attrition among various ownerships.

### 2.4 Statistical simulation of forest loss

Forest loss and the resulting attrition are usually spatially correlated. For example, fire and disease usually start from an origin point and gradually affect contagious forests near the origin. Logging activities usually concentrate in a continuous part of forestlands for the purpose of cost-efficiency, meaning that forest losses in this case are highly organized and spatially correlated. In order to assess the statistical significance of forest attrition, we incorporated the effects of both spatial randomness and spatial correlation in the process of forest loss.

By subtracting forest maps of a later time from those of the initial time we generated binary maps of forest loss with the presence of forest loss as 1 pixels and absence of forest loss as 0 pixels. The patches of forest losses were delineated in a forest loss cluster map as contagious loss pixels based on their spatial connectivity using 8-neighborhood rule. Only forest losses were considered. From the forest loss cluster maps, a frequency distribution was created expressing the count of clusters with respect to their sizes. The histogram acted as a constraint for the simulations of forest loss.

While the simulated forest loss patches were constrained to the same size and count of forest loss clusters in the observed case, the shapes of forest loss clusters were not constrained as long as forest loss pixels are spatially connected. These random forest loss shapes were generated by expanding around a single forest loss pixel randomly in 8 directions until a target size was reached. In doing so, forest loss simulation processes were constrained for both quantity and spatial autocorrelation by keeping the same pixel count and spatial continuity as the observed forest losses, because many disturbances tend to be spatially clustered and contiguous, e.g., fire, insects, harvesting, etc. These steps were applied 1000 times in each level III ecoregion [77] thus generating 1000 forest change maps (See S3 Fig for level III ecoregion boundaries and distributions). Mosaicking of all ecoregions followed to create 1000 change maps for the conterminous U.S. Forest attrition distance metrics were calculated using these 1000 continental maps. Ecoregion names and basic forest statistics are available in S1 Table.

## Results

### 3.1 Spatial distribution of forest cover and forest attrition dynamics

**Fig 2:**
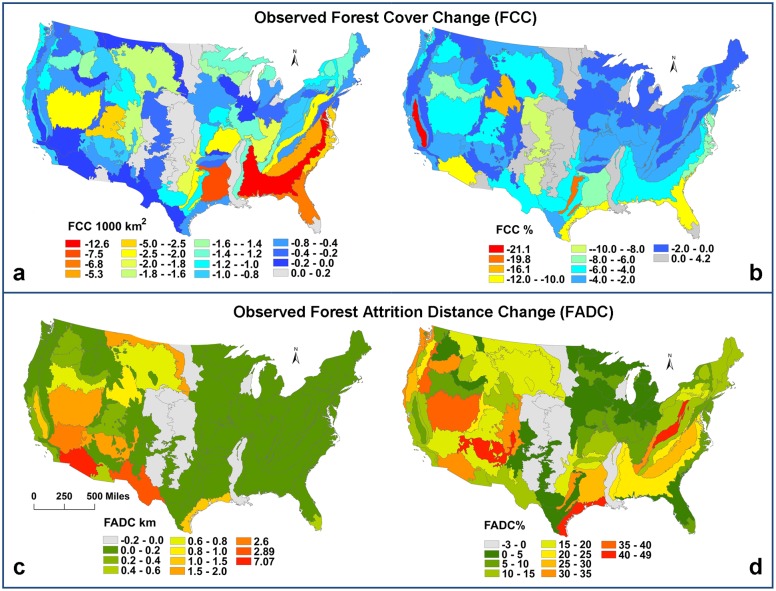
Forest cover change (FCC) and forest attrition distance change (FADC) in level III ecoregions. While the southeastern U.S. is experiencing high forest loss, the highest forest attrition is concentrated in other parts of the country.

Spatial representations of the forest cover and forest attrition distance dynamics are presented in Fig 2. Forest cover change (FCC) is calculated by subtracting the amount of forest in 1992 from the amount of forest in 2001(Fig 2a). Negative values in red and orange legend classes represent large amount of forest loss in an ecoregion. These large forest losses (>3000km^2^) are distributed along the south east coast, which is consistent with the previous work. For example, it was shown that forest disturbance rates are highest in the southeastern U.S. near northwestern Louisiana and eastern Texas due to softwood forestry during the same ten year period [78], which is consistent with Fig 2a. Other studies of continental U.S. forest cover changes in the 1990s also showed similar pattern in southeastern U.S. [72,79–81]. The percentage of forest cover change (FCC%) depicted in Fig 2b in red for negative values while grey classes represent forest gains in ecoregions. Severe losses occurred around the Gulf of Mexico Coast, along the coast near California, and around northwest parts of U.S. Furthermore, observed forest distance changes (FADC) are calculated by subtracting forest attrition distances in 1992 from forest attrition distances in 2001 in ecoregions (Fig 2c). Positive values are in red and orange. Most ecoregions (79 out of 85) have observed forest attrition distance increases. A number of ecoregions with high forest attrition distance increases cluster in the southwest. A few ecoregions in the Great Plains and around the Mississippi River reduced their forest attrition distances. In Fig 2d, the observed forest attrition distance change percentage (FADC%) is calculated as the proportion of the forest attrition distance change to the forest attrition distance in 1992. Legend classes of high positive percentage (red color) represent large forest attrition distance increases from 1992 to 2001, namely high forest attrition. Several regions with high forest attrition distance change (> 30%) occur in West Virginia, Kentucky, and North Carolina. These ecoregions showed relatively low forest cover loss (< 4%) in Fig 2b. Similarly, a few ecoregions along the Oregon-Washington coast show high percentage (>30%) of forest attrition distance increases yet low forest cover loss (<3%). Similar cases occur in Arizona, Colorado, and Nevada with over 30% forest attrition distance changes and less than 5% forest cover loss. All these cases are examples of accelerated forest attrition patterns.

### 3.2 Statistical significance of forest attrition dynamics

At the national level the total forest cover loss was approximately 90,400 km^2^ (34,900 square miles), approximately the size of the state of Maine, constituting a relative change of -2.96%. Examining the spatial arrangement of this change the average FAD was 3674m in 1992 and increased by 514m or 13.99% in 2001. Simulations based on the same amount and patch size of forest cover loss indicated only a 10m FAD increase suggesting that the observed FAD increase was more than an order of magnitude higher than expected.

**Fig 3:**
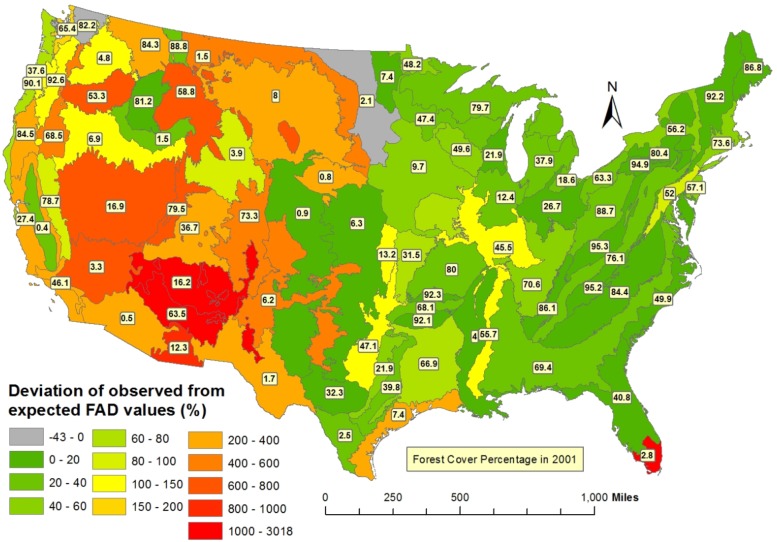
Deviation of observed from expected FAD values (%). Yellow labels depict percentage forest cover in 2001. High deviation values concentrate in Pacific Northwest ecoregions and the Southwest coast suggesting more severe clustering of forest losses and attrition around those regions.

To examine further the spatial distribution of the FAD significance results were segmented into 84 ecoregions in the continental U.S. for the years 1990 and 2000 (individual ecoregion values are available in S1 Table). For each ecoregion the normalized percent change FAD (FADC_Norm%_) was computed comparing the percent change of FAD values resulting from the observed and the mean simulated FAD values (Fig 3). Positive FADC_Norm%_ indicate losses of isolated forest patches while negative values suggest dispersed and encroaching forest losses. In 78 out of 84 ecoregions the observed FAD values in 2001 were higher than +3 standard deviations above the simulated expected mean FAD values suggesting disproportionate forest attrition for their corresponding observed forest cover loss. High FADC_Norm%_ values are concentrated in the Pacific Northwest ecoregions and southwest coast, including both highly forested ecoregions (>60%) and less forested ecoregions (<10%).

The FADC_Norm%_ values also exhibit high variability across ecoregions. This can lead to valuable insights for planning efficient and effective management efforts. Among the 84 ecoregions, 11 out of 40 cases of severe attrition increases (FADC_Norm%_ >50) occurred in highly forested (>60%) ecoregions (Fig 3, the 11 ecoregions are marked by asterisks in S1 Table). This is surprising because highly forested ecoregions tend to have more continuous forest cover and larger forest patches where forest attrition is less likely to happen [39]. Therefore, the attrition and resulting removal of larger forest patches in these regions is indicative of extensive forest disturbances possibly featuring spatially-continuous clearcutting, clustered disease and pest outbreaks and large natural disasters.

### 3.3 Establishing a relationship between forest cover and forest attrition

**Fig 4:**
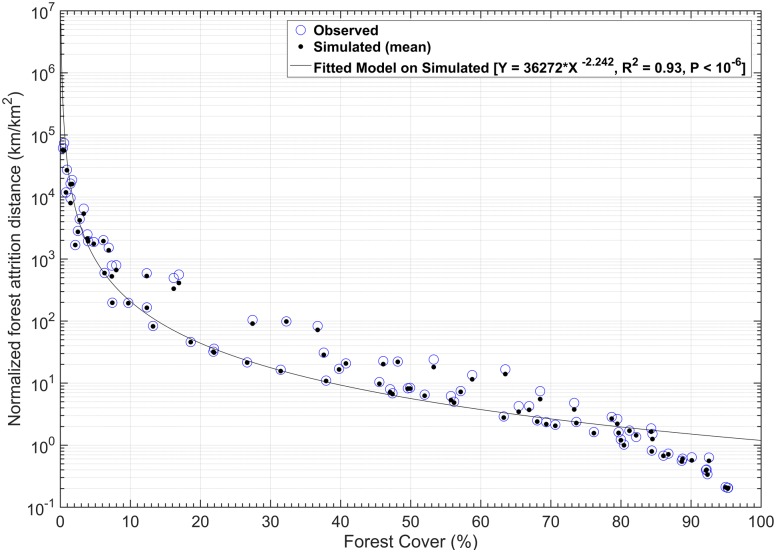
Scatterplot of forest cover percentage and forest attrition distance normalized per forest cover. Data generated from observations and statistical simulations from 84 level III ecoregions in 2001. The accurate (R^2^ of 0.93) nonlinear relationship suggests accelerated attrition as percentage forest cover decreases and allows prediction of attrition increases given certain forest cover changes.

In order to further investigate the behavior of FAD under different forest cover, the simulated FAD values were first normalized by forest cover for better comparison among ecoregions and then plotted against forest cover percentage (Fig 4). A mathematical model between forest attrition and forest cover is established (y = 36272*x ^-2.242^, R^2^ = 0.93). The nonlinear relationship suggests monotonic and accelerated attrition as percentage forest cover decreases and allows prediction of expected attrition increases given certain forest cover changes. Positive deviations from the model suggest forest losses tend to be clustered disappearance of entire patches, while values below the line, or negative deviations, indicate dispersed encroachment similar to forest perforation.

### 3.4 Forest attrition distribution across urbanization and landscape ownership strata

**Fig 5:**
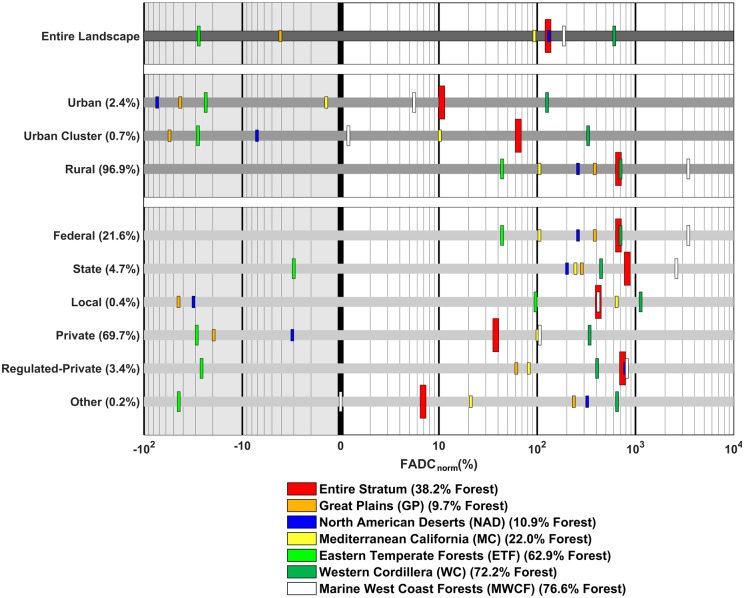
FAD_Norm_ dynamics in landscape strata over six ecoregions. FADC_Norm_ values are depicted in bars. Percentage of land cover strata is shown in parentheses next to the name. Percentage of forest cover in ecoregions is shown in parentheses in the legend. Forest attrition is considerably higher in western ecoregions and federal and state lands while lower in urban and suburban regions.

We further studied forest attrition in two contextual landscape segmentations: i) urbanization levels to more closely assess forests’ direct human benefits such as providing sources of fresh water [82], purifying air and reducing storm runoff [83], preventing erosion and damage to roads and structures [84], dampening the effects of urban heat island [85,86], and reducing greenhouse gas emission [87–90]because urban areas represent immediate human influence and direct land conversion pressure from urban expansion and ii) land ownership types as forest ownership type impacts forest disturbance [91], forest landscape pattern and animal habitat [92–94], stand structure and carbon storage [95], forest aboveground biomass and landscape dynamics [96]. Forest attrition dynamics in each landscape stratum are reported in Fig 5. Results are further separated in six major ecoregions with different densities of forest cover.

Looking at the entire landscape (first row in Fig 5) arguably the most worrisome ecoregions with high percentage forest cover are in the west (WC and MWCF ecoregions) where the corresponding deviation from the expected normalized FAD change value is above 100%, compared with <-11% in the eastern ETF ecoregion. The expected value is calculated based on the relation between forest cover change and normalized FADC established in the previous section. This difference could be attributed to attrition taking place within these two western ecoregions but also loss of key forests in adjacent ecoregions that serve as their closest resource. There is also high variability between the low percentage forest cover ecoregions. Observed attrition is substantially higher than expected in the NAD (127.6%) than in the GP region (-4.5%). This difference is despite the fact that both overall forest cover and relative forest change cover are similar in value.

Detailed FAD data for the 84 level III ecoregions are available in S2 and S3 Tables.

Examining distribution of forest attrition at different urbanization levels (Fig 5, rows 2–4) forest attrition is significantly lower than expected in urban and suburban regions compared to the rural landscape strata. This lower attrition is manifested more intensely in ecoregions with low percentage forest cover, NAD and MC. In addition, within the two urban strata, attrition is much higher than expected in western forests (WC ecoregion, 125.9% for urban and 329.7% for urban cluster strata) than eastern forests (ETF ecoregion, -23.58% for urban and -28.3% for urban cluster strata), despite similar forest cover loss. Beyond the normalized forest attrition it is also worth reporting the significantly higher forest cover losses in urban and suburban ecoregions, which are typically 3–4 times higher percentagewise than in rural areas. This indicates scattered forest losses and limiting forest benefits on biodiversity and ecosystem, e.g., for carbon storage and air quality improvement [97]. Perhaps, urban/suburban areas may have had management practices that greatly mitigated attrition effects whereas such management is lacking in rural areas.

Land ownership is also exhibiting wide differences in observed forest attrition versus expected values (Fig 5, bottom 6 rows). Overall, federal and state ownership experiences more than 20 times the attrition of private lands. Typically, forest management on private lands receives significant attention due to the magnitude of forest loss—forest cover loss is 3–4 times higher on private lands than federal and state lands. However, the spatial arrangement of the forest loss on federal and state lands indicates that despite low magnitude forest loses, these loses could be of potentially higher environmental importance. Alternatively, these public lands are more susceptible to forest losses taking place in adjacent private lands. High forest attrition is also driven predominately from the western high density forested ecoregions (WC, MWCF).

## Discussion

The presented decadal analysis of forest attrition across US landscapes offers several advancements in landscape transformation. FAD supports attrition monitoring and consistent temporal comparison of forest pattern dynamics, which is critical in managing forests under increasing human and environmental pressures. There are numerous impacts of forest attrition including complete habitat losses, severe decline of population sizes and species richness, and shifts of local and regional environmental conditions.

The spatial arrangement of isolated forest loss requires higher conservation priority compared to other spatial processes, e.g., subdivision and fragmentation, which cause indirect losses for certain species [98]. High attrition in extensively forested ecoregions poses warnings for forest conservation and restoration. It also suggests that the widely-accepted model of forest transformation [39]can be skewed where perforation and fragmentation should have been dominant spatial processes in high forest percentage areas. In addition, for ecoregions with lower forest cover percentage, forest attrition frequently removes smaller patches which are keystone structures providing microclimate, soil nutrients, plant species, and animal habitat connectivity at local and landscape levels [99,100].

**Fig 6:**
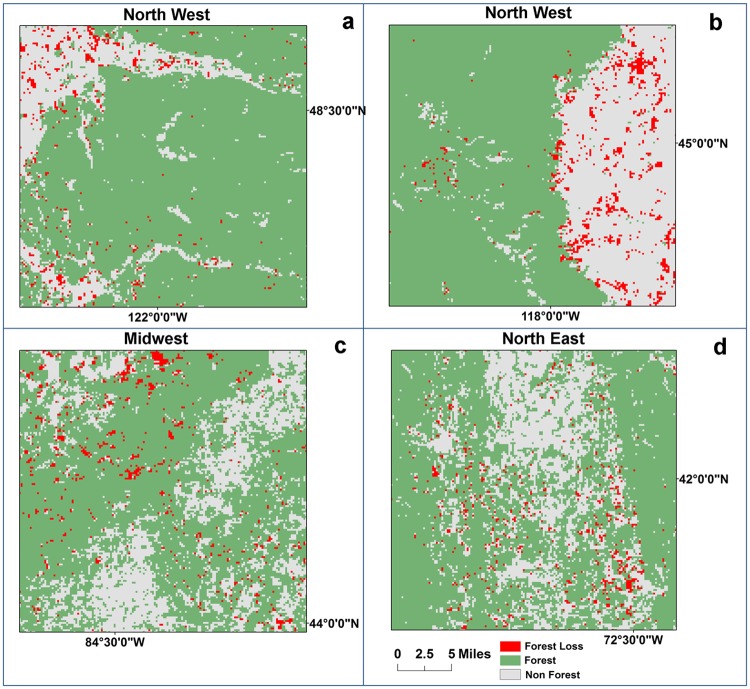
Forest attrition visualized in four ecoregions. Forest losses during the 1990s are depicted as red pixels, forest present in 2000 is shown as green pixels and non-forest is shown as gray pixels. Forest attrition is considerably higher in western ecoregions than in eastern ecoregions as forest losses occurred more frequently in gap regions.

Among the skewing factors are urbanization levels and land ownership. While urban areas have received most of the attention, our study shows that it is the rural areas that suffer higher attrition levels. Furthermore, it is the federal and state lands that exhibit higher attrition, necessitating improvements in public land management. A visual example showcasing the differences between eastern and western regions is depicted in Fig 6. Forest losses are dominant in gap areas in western ecoregions leading to severe forest attritions, whereas in eastern regions forest losses appear in the interior or near the forest edge thus causing lower attrition. This difference may be attributed to many factors including lower tree density and higher terrain heterogeneity in western than eastern ecoregions. In addition, the predominant disturbance classes are fire and insect activities in the west compared to harvesting in managed forests in the east [81], possibly leading to different attrition patterns in these two strata.

From the biodiversity conservation perspective changes in climatic conditions, such as temperature and precipitation, can be projected after forest attrition is estimated. Estimating these climatic changes can lead to better understanding of potential impacts on genetic diversity of various species [41]. In addition, in order to promote carbon sequestration in forests and in turn mitigate climate changes, biomass and carbon changes from forest attrition need to be carefully evaluated to determine most profitable mitigation measures, for example reforestation [46], because forest attrition often causes irreversible carbon losses compared to other geographic patterns of forest loss. Finally, the locations of primary forests in more pristine condition or scattered forest patches serving as keystone structures can be overlaid with the forest attrition maps for informing forest management planning. This is especially critical in conserving climate, biodiversity, soil nutrient, and microclimate [43,99].

In terms of conceptual advancements several indicators have been proposed in the past [27–29], however they fail to support multi-temporal, multi-scale comparisons while being independent of study boundaries. In contrast, the proposed proximity-based aggregation indicator is independent of spatial scale and extent, thus supporting from detailed local to coarse global applications. The effectiveness of the resampling process is also partly related to the insensitiveness of the metric to spatial scales. We should note that our method, like any spatial analysis of land cover products, is dependent on the definition of the minimum mapping unit. In our study we strived for a balance between reliable information and ecologically meaningful analysis. We chose the 1 ha as the minimum mapping unit following internationally accepted protocols from the United Nationals Framework Convention on Climate Change (UNFCCC). We also removed very small patches as potential map classification errors. Analysis with different assumptions could lead to different results so interpretation of our findings should consider our initial assumptions and intent to provide a national rather than a localized comparison.

On the other hand, a considerable advantage is our metric’s independent of spatial scale in terms of spatial extent of application, because FAD can be applied and aggregated from local to global scales without the constraints of boundary and size of study areas. Furthermore, since our method does not directly calculate patch statistics that are challenging to generalize over time (e.g. patches may be lost or added) it offers a consistent comparison across time periods. From the algorithmic implementation and interpretation, our method is intuitive for non-experts as the underlying calculation is a straightforward distance calculation. The methodology is straightforward to implement and numerous geospatial software packages have embedded the needed functionality.

To summarize, our hope is that this easy to use metric alongside with the obtained results would help balance development and conservation needs in a more effective way without losing biodiversity and carbon sequestration capabilities. Adding spatial pattern context behind general forest dynamic metrics is an important step towards this direction. Our analysis covered the continental U.S., however there are no barriers towards a global application beyond computation power (for example using the recently produced global tree cover maps by [64]). While our results offer important information it is important to pursue understanding of the processes behind these patterns, an essential step for proactive conservation management.

## Supporting information

S1 Fig Urban and rural areas in continental U.S. (TIF)

S2 Fig Boundaries of major ownership lands in continental U.S. (TIF)

S3 Fig EPA level I and level III ecoregions in continental U.S. (TIF)

S1 Table Statistics of forestlands in level III ecoregions. (XLSX)

S2 Table Statistics of forestlands in urban/rural areas in level III ecoregions. (XLSX)

S3 Table Statistics of forestlands in 5 ownership areas in level III ecoregions. (XLSX)
