# Supplementary figures and images for: Forest dynamics in the U.S. indicate disproportionate attrition in western forests, rural areas and public lands
Source: PLoS One. 2017 Feb 22;12(2):e0171383. doi: 10.1371/journal.pone.0171383 (PMC5321268; doi:10.1371/journal.pone.0171383)

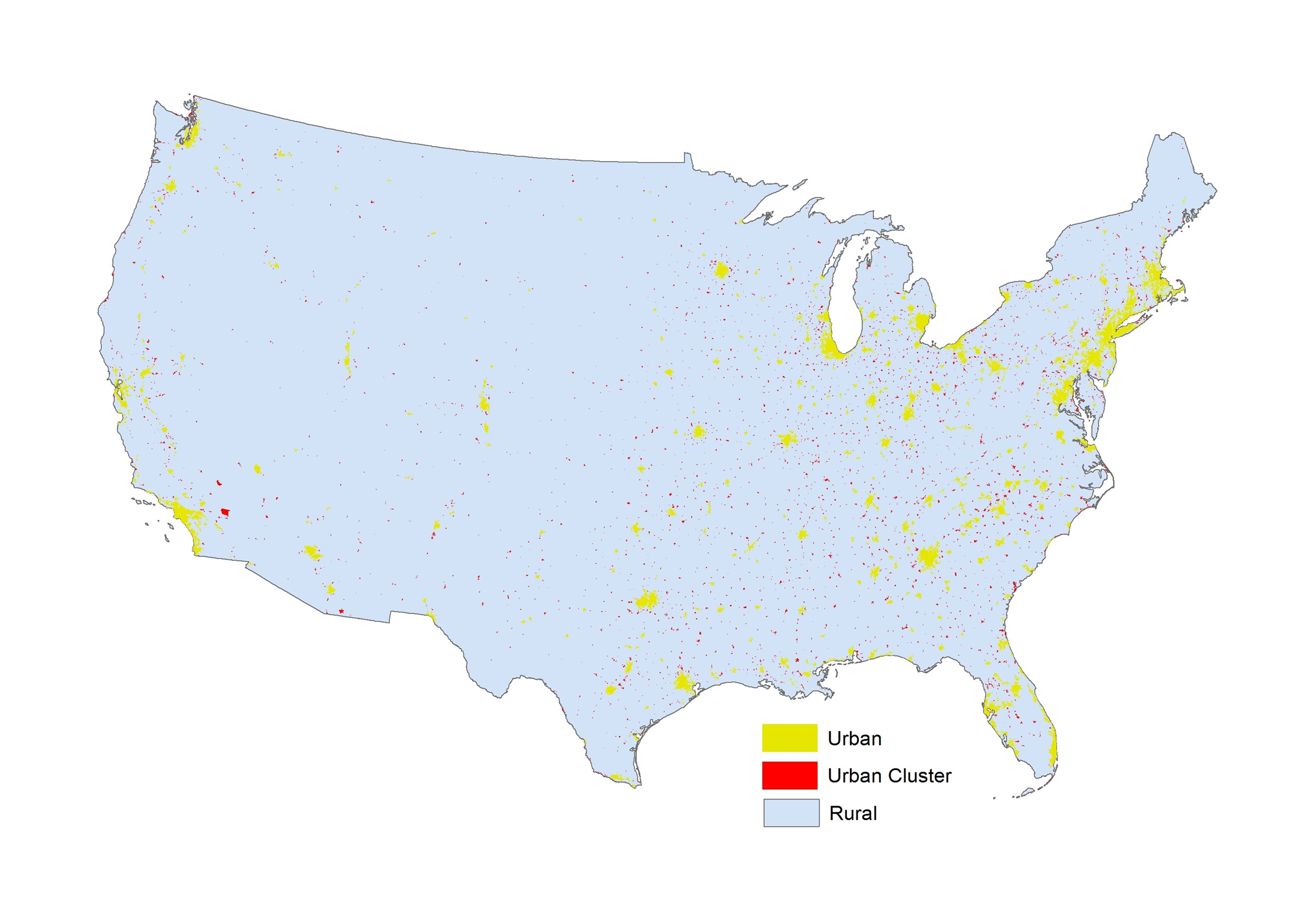

Supplement: S1 Fig — (TIF) [file pone.0171383.s001.tif]

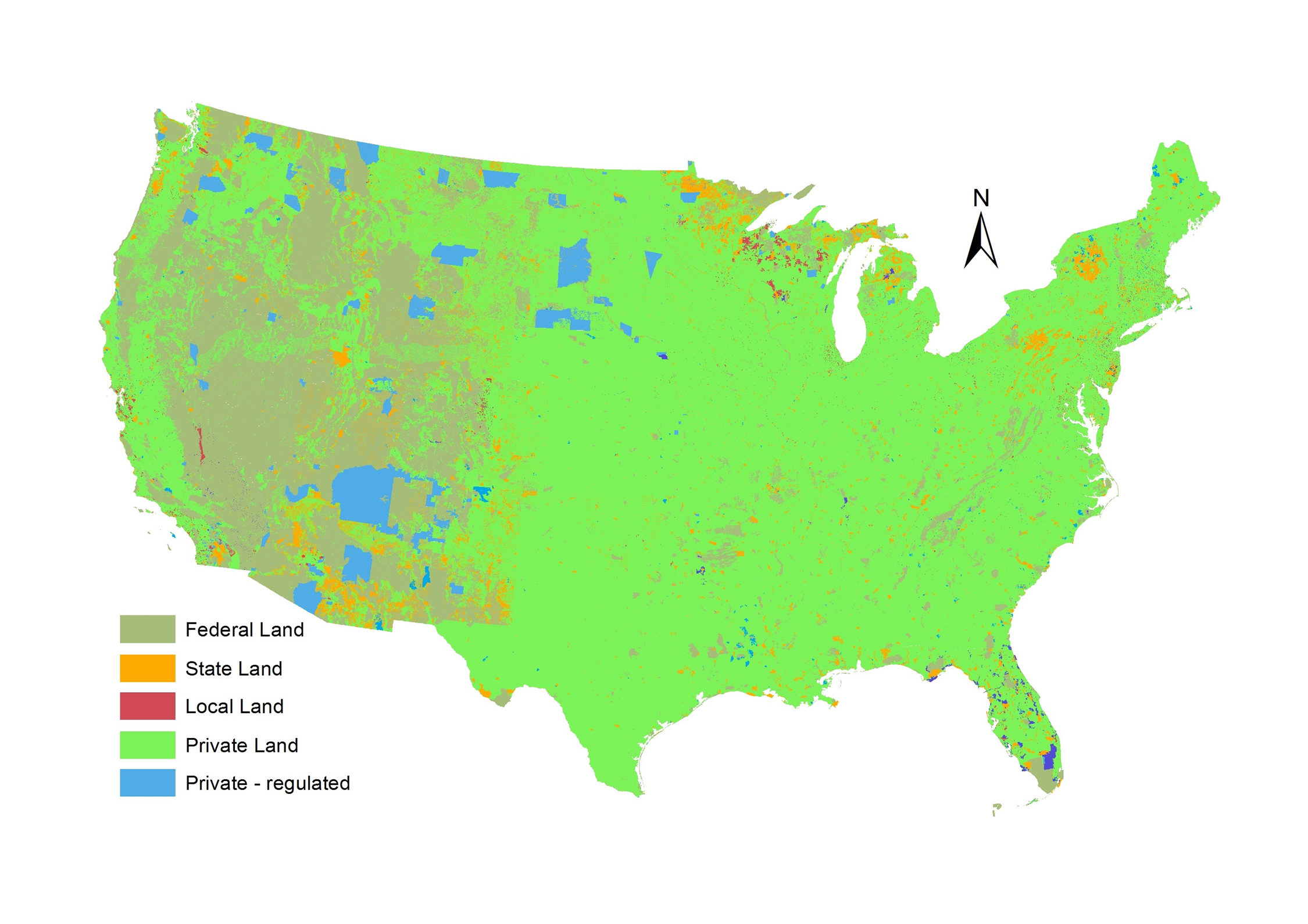

Supplement: S2 Fig — (TIF) [file pone.0171383.s002.tif]

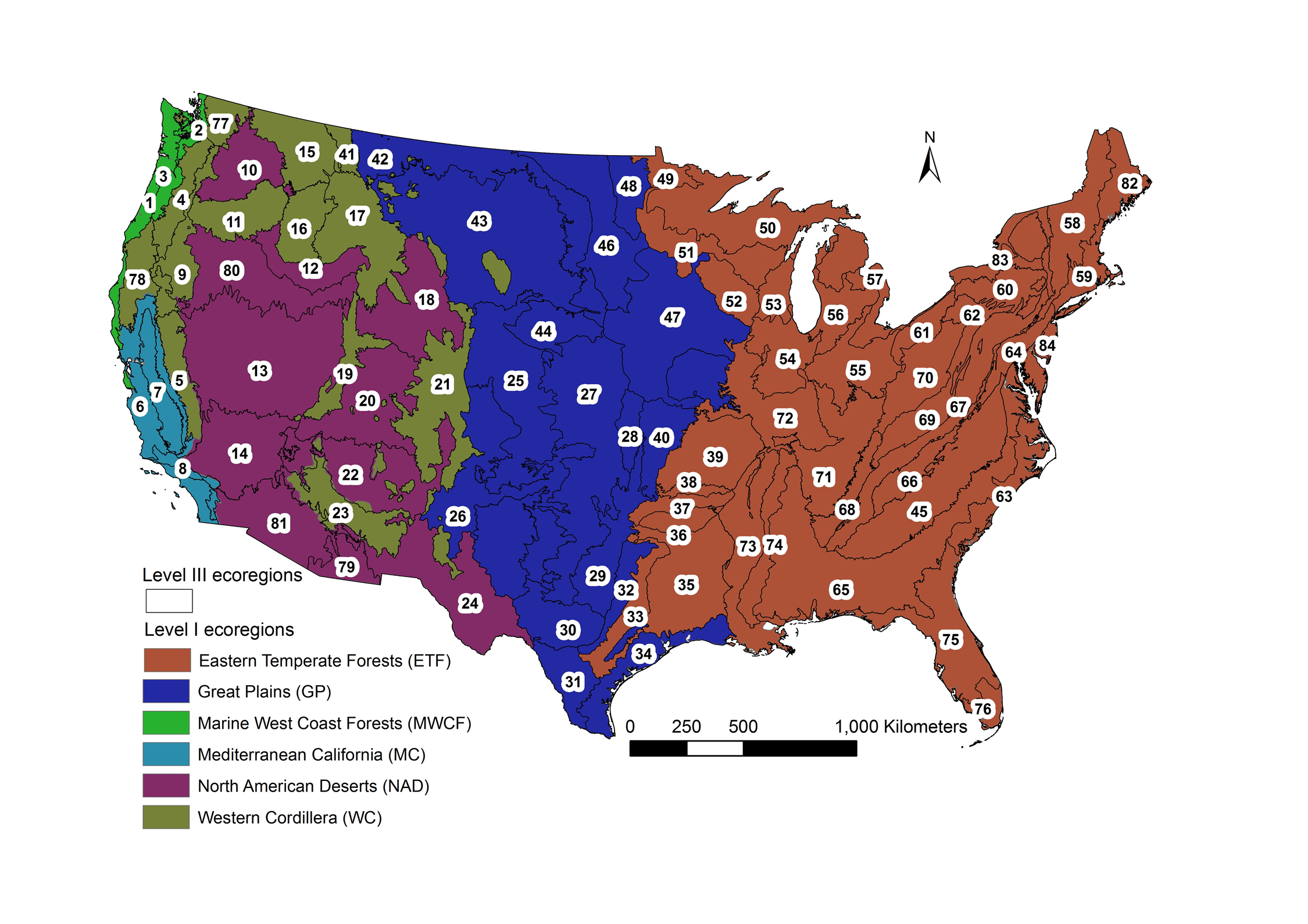

Supplement: S3 Fig — (TIF) [file pone.0171383.s003.tif]
